# Hyperglycemia Impairs Neutrophil-Mediated Bacterial Clearance in Mice Infected with the Lyme Disease Pathogen

**DOI:** 10.1371/journal.pone.0158019

**Published:** 2016-06-24

**Authors:** Ashkan Javid, Nataliya Zlotnikov, Helena Pětrošová, Tian Tian Tang, Yang Zhang, Anil K. Bansal, Rhodaba Ebady, Maitry Parikh, Mijhgan Ahmed, Chunxiang Sun, Susan Newbigging, Yae Ram Kim, Marianna Santana Sosa, Michael Glogauer, Tara J. Moriarty

**Affiliations:** 1 Matrix Dynamics Group, Faculty of Dentistry, University of Toronto, Fitzgerald Building, Room 241, 150 College Street, Toronto, Ontario, M5S 3E2, Canada; 2 Mount Sinai Hospital/Research Institute, The Toronto Centre for Phenogenomics, 25 Orde Street, Toronto, Ontario, M5T 3H7, Canada; University of Kentucky College of Medicine, UNITED STATES

## Abstract

Insulin-insufficient type 1 diabetes is associated with attenuated bactericidal function of neutrophils, which are key mediators of innate immune responses to microbes as well as pathological inflammatory processes. Neutrophils are central to immune responses to the Lyme pathogen *Borrelia burgdorferi*. The effect of hyperglycemia on host susceptibility to and outcomes of *B*. *burgdorferi* infection has not been examined. The present study investigated the impact of sustained obesity-independent hyperglycemia in mice on bacterial clearance, inflammatory pathology and neutrophil responses to *B*. *burgdorferi*. Hyperglycemia was associated with reduced arthritis incidence but more widespread tissue colonization and reduced clearance of bacterial DNA in multiple tissues including brain, heart, liver, lung and knee joint. *B*. *burgdorferi* uptake and killing were impaired in neutrophils isolated from hyperglycemic mice. Thus, attenuated neutrophil function in insulin-insufficient hyperglycemia was associated with reduced *B*. *burgdorferi* clearance in target organs. These data suggest that investigating the effects of comorbid conditions such as diabetes on outcomes of *B*. *burgdorferi* infections in humans may be warranted.

## Introduction

Lyme disease, also known as Lyme borreliosis, is the most common vector-borne disease in temperate climates, with an estimated annual incidence of ~300,000 cases in the United States [1]. Lyme disease is caused by members of the *Borrelia burgdorferi* sensu lato species complex, which are transmitted to humans and other vertebrate hosts during the blood meal of infected ticks. Following tick transmission, *B*. *burgdorferi* disseminates widely through the host and colonizes multiple organs and tissues [2–4]. Clinical manifestations of Lyme disease include erythema migrans, arthritis, carditis, and neuroborreliosis; the majority of these manifestations are thought to result from pathological host immune responses to infection, rather than damage caused by *B*. *burgdorferi* [5,6]. *B*. *burgdorferi* can persist for extended periods in the host, likely as a result of reduction in bacterial burden to levels that do not elicit host-damaging immune responses, changes in the nature of immune responses to bacteria, and/or gradual resolution of inflammatory pathology [7]. Thus, factors that alter immune responses to *B*. *burgdorferi* infection and bacterial burden in tissues can affect disease progression and outcomes.

Lyme disease incidence has been increasing in North American and European countries, where the prevalence of diabetes is also growing [1,8]. Both Type I (insulin-insufficient) and Type II (obesity-associated insulin-resistant) diabetes increase susceptibility to infection with many pathogens and worsens infection outcomes in animal models and humans, most notably for *Staphylococcus aureus*, *Burkholderia pseudomallei* and *Mycobacterium tuberculosis* infections [9–13]. An important feature of aberrant immune responses in obesity-independent hyperglycemia is neutrophil dysfunction. Hyperglycemia dysregulates neutrophil activation and impairs the ability of neutrophils to phagocytose and kill *S*. *aureus*, *B*. *pseudomallei*, and non-pathogenic *Escherichia coli in vitro*, and to control *S*. *aureus* infection *in vivo* [13–18]. Neutrophils are important contributors to both innate host defense against *B*. *burgdorferi* and inflammatory pathology in Lyme disease. They are recruited to the heart and joints early in infection and are a major constituent of inflammatory infiltrates in Lyme arthritis, where they contribute to immunopathology and control bacterial burden [19–24].

Collectively, these observations prompted us to examine whether obesity-independent hyperglycemia caused by insufficient insulin levels (Type 1 diabetes) alters the outcomes of *B*. *burgdorferi* infection in mouse models of Lyme disease. To determine if hyperglycemia influences *B*. *burgdorferi* infection and its disease outcomes, we investigated the effects of obesity-independent hyperglycemia on *B*. *burgdorferi* clearance and dissemination, disease severity, and bacterial uptake and killing by neutrophils in mouse models of Type 1 diabetes and Lyme disease.

## Material and Methods

### Ethics Statements

This study was carried out in accordance with the principles outlined in the most recent policies and *Guide to the Care and Use of Experimental* Animals by The Canadian Council on Animal Care. All animal work was approved by the University of Toronto Animal Care Committee in accordance with institutional guidelines (Protocol 010430). Work with *B*. *burgdorferi* was carried out in accordance with University of Toronto, Public Health Agency of Canada, and Canadian Food Inspection Agency guidelines (University of Toronto biosafety permit 12a-M30-2). The authors declare that there are no conflicts of interest.

### Animals

Male C57BL/6NCrl and C3H/HeNCrl mice purchased from (Charles River, Montréal, QC) and male heterozygote C57BL6J-Ins2^Akita/J^ mice (Jackson Labs, Sacramento, CA) were housed in groups of 3 or 4 per cage under pathogen-free conditions with environmental enrichment, and fed *ad libitum* throughout experiments with standard rodent chow (Tekland 2018 Rodent Chow, Harlan Laboratories, Mississauga, ON). Equal numbers of mice were randomly assigned to experimental and control groups upon arrival. Mice were monitored daily throughout experiments for lethargy, weight loss, failure to groom, lameness and dehydration.

### Breeding, genotyping and phenotyping of Akita mice

C57BL6J-Ins2^Akita/J^ male mice were crossed with C57BL/6J female mice (Jackson Labs) bred in-house to generate heterozygous Ins2^Akita^ mice. DNA for genotyping was extracted from tail snips using the Qiagen DNeasy tissue extraction kit, following manufacturer’s instructions (Qiagen, Toronto, ON). PCR genotyping was performed by amplification using primers oIMR1093 (5’-TGCTGATGCCCTGGCCTGCT-3’) and oIMR1094 (5’-TGGTCCCACATATGCACATG-3’), followed by overnight digestion of PCR products with Fnu4HI. Digestion products were resolved on 3% agarose gels to visualize DNA bands corresponding to wild-type (140 bp) and mutant (280 bp) gene sequences. Hyperglycemia phenotyping was performed by measuring non-fasting blood glucose in blood obtained from the saphenous vein using a calibrated Aviva Nano glucometer and glucose strips (Accu-Check/Roche, Laval, QC). Due to variable time to onset of the hyperglycemia phenotype in Akita mice, infections were performed in age-matched animals ranging from 4 to 8 weeks of age. Control mice for Akita experiments were normoglycemic homozygous wild-type littermates. Characteristics of all mouse strains used in this study are summarized in Table 1.

**Table 1:** Mouse strains used in this study.

Mouse strain	Model of	Lyme carditis	Lyme arthritis	Induction of hyperglycemia	Age at the time of infection
C57BL/6	Diabetes and obesity	R^*a*^	R^*a*^	5 days of STZ treatment	6–7 weeks
C3H/HeN	*B*. *burgdorferi* infection	S^*a*^	S^*a*^	7 days of STZ treatment	6–7 weeks; 4–5 weeks for arthritis studies
Akita	Hyperglycemia	Likely R due to C57 background (NA^*b*^)	Likely R due to C57 background (NA^*b*^)	Spontaneous, due to a dominant-negative mutation in the *ins2* gene	4–8 weeks, due to variable time to onset of the hyperglycemia phenotype

^*a*^ R and S stand for resistant and sensitive, respectively [25].

^*b*^ NA–information not available.

### STZ induction of hyperglycemia

Five-week-old male mice (all experiments except for arthritis studies) or 3-week-old male C3H/HeN mice (arthritis studies) were rendered diabetic by a multiple low-dose streptozotocin (STZ) treatment as described previously [26]. Briefly, mice fed *ad libitum* were injected intraperitoneally with either 40 μg STZ (Cedarlane Laboratories, Burlington, ON) per gram body weight in 0.1 M sodium citrate or equal volume of buffer without STZ (vehicle) once daily for 5 (C57BL/6) or 7 (C3H/HeN) consecutive days. Mice were considered hyperglycemic when non-fasting blood glucose levels reached 15 mmol/L. Non-fasting blood glucose was >25 mmol/L in all STZ-treated mice at time of infection and sacrifice. Non-fasting blood glucose in blood obtained from the saphenous vein was measured using a calibrated Aviva Nano glucometer and glucose strips (Accu-Check/Roche). STZ-treated animals were fed semi-liquefied mash of standard diet to prevent dehydration as previously described [27]. Blood glucose and body weight were measured in all animals before experimental treatment, every 2–3 days during STZ treatment, on the day of infection with *B*. *burgdorferi* and at the time of sacrifice.

### *Borrelia burgdorferi strains*, cultivation and mouse infections

Infections were performed with freshly inoculated cultures of log phase GCB726, a B31 5A4 NP1-derived infectious strain of *B*. *burgdorferi* transformed with GFP-expressing plasmid pTM61 [28]. Cultures were grown in Barbour-Stoenner-Kelly-II (BSK-II) medium prepared as previously described [29] supplemented with 6% heat-inactivated rabbit serum (Cedarlane Laboratories) and 100 μg/mL gentamycin (Bioshop Canada Inc, Burlington, ON) at 36°C and 1.5% CO_2_.

Five days after the last STZ treatment (“washout period”), mice from vehicle- and STZ-treated groups received a subcutaneous injection at the dorsal lumbar midline with either 1x10^4^ *B*. *burgdorferi* suspended in BSK-II medium or with BSK-II medium alone (mock-infected control). For all experiments except for arthritis studies, C57BL/6 and C3H/HeN mice were 6–7 weeks of age at time of infection. We used 6–7 week-old animals in most experiments because STZ treatment disrupts weight gain during maturation to adulthood and can be lethal in young mice [30] at the age typically used for *B*. *burgdorferi* infectivity studies (3–4 weeks). For arthritis studies, mice were less than 5 weeks of age at time of infection. Due to variable time to onset of the hyperglycemia phenotype in Akita mice [31], infections in heterozygotes and homozygous wild-type normoglycemic controls were performed in age-matched animals ranging from 4–8 weeks of age.

At 4 weeks post-infection, non-fasting peripheral blood glucose and body weight were measured, animals were then anesthetized with 2% isoflurane, and blood was drawn by cardiac puncture for complete blood count (CBC) analysis. Animals were then euthanized by cervical dislocation and tissues and neutrophils were harvested for histology, quantitative real-time PCR (qPCR), and *in vitro* neutrophil function assays.

### DNA extraction and qPCR for measurement of bacterial DNA copy number

Total DNA concentration and qPCR determination of bacterial burden were measured in blood, brain, bladder, ear, heart, liver, lung, knee joint (patella), and ventral thoracic skin harvested from animals. Total DNA was extracted using the Qiagen DNeasy tissue extraction kit, following manufacturer’s instructions. Concentration and purity of extracted DNA were measured using a NanoDrop spectrophotometer (Thermo Fisher Scientific, Toronto, ON). qPCR measurement of *flaB* DNA copy number was performed as described previously [32,33] using a CFX96 real-time PCR machine (Bio-Rad Laboratories, Mississauga, ON). Briefly, each qPCR assay was performed with duplicate standards containing 10^1^–10^6^ copies of plasmid pTM222 encoding the *flaB* segment for qPCR amplification on the same plate as sextuplicate reactions containing DNA extracted from each tissue sample. Reactions were performed in 1X iQ SsoFast EvaGreen Supermix (Bio-Rad) prepared according to manufacturer’s instructions and contained 400 nM of each of *flaB* primers T1 (5'-GCAGCTAATGTTGCAAATCTTTTC-3') and T2 (5'- GCAGGTGCTGGCTGTTGA-3'). qPCR was performed using 2 μl of extracted DNA, in a total reaction volume of 20 μl. PCR conditions: Step 1: 98°C 2 min; Step 2: 40 cycles of 98°C 5 s, 59.2°C 5 s; Step 3: 65°C 5 s; Step 4: melting curve analysis over melting range 65°C to 95°C. Standards were used to calculate the exact number of copies of *flaB* sequence in samples. Each plate included negative control wells (DNA extraction elution buffer). The R^2^ value of the standard curve obtained on every run was examined to ensure run quality and pipetting accuracy; runs with R^2^ values below 0.85 were repeated. The average copy number obtained from all qPCR repeats for each sample was used in subsequent graphing and statistical analysis. Copy numbers from samples with aberrant melting or amplification curves compared to replicate measurements for the same sample were not included in calculations of average copy number. *flaB* DNA copy numbers for samples were normalized to total DNA concentration (copy number per μg of DNA) to control for differences in DNA extraction efficiency among samples. Tissues were considered qPCR-positive if at least one *flaB* DNA copy was detected per μg of total DNA isolated from tissues.

### Histology, arthritis and carditis scoring

Sagittally hemisected heart samples were harvested from sacrificed animals. Both the right and left tibiotarsal ankle joints (with skin) were excised using forceps (with a few millimeters left both above and below the joint) and placed immediately in 1.5 ml 10% neutral-buffered formalin (Sigma Chemicals, St Louis, MO), which was changed after 24 hours of fixation [34]. Samples were embedded in paraffin, sectioned, and stained with hematoxylin and eosin (H&E) by the histology services of the University of Toronto Faculty of Dentistry, Hospital for Sick Children and Toronto Centre for Phenogenomics. Tibiotarsal joints were decalcified for 96 hours in TBD-2 Decalcifier solution (Thermo-Fisher Scientific), prior to embedding, sectioning and H&E staining. The stained tissue sections included the tibiotarsal joint and were sectioned longitudinally with view of all long bones and joints. A veterinary pathologist analyzed tissue sections in a blinded fashion under light microscopy for any abnormal lesions. A score of 0, 1 or 2 was assigned to each section based on severity of inflammation in the joints, and corresponded to: no significant pathological findings (0), mild pathology (a few neutrophils within the joint space and large basophilic ‘smudge’-like particles) (1), and severe pathology (large numbers of neutrophils within the joint space, large basophilic ‘smudge’-like particles and extension of inflammation into the subcutaneous tissue around the joint) (2).

Scoring of inflammation in hearts was performed by modification of a previously described protocol for quantifying multifocal cardiac inflammation [35]. Briefly, the number of nuclei in five 100 mm^2^ regions of interest in 2–3 matched H&E-stained sagittal sections per heart were enumerated using a counting grid and the average number of nuclei per region of interest in each section was calculated. Nuclei were counted in each atrium and ventricle and the heart apex. The majority of tissue included in each region of interest was derived from the myocardium.

### Complete Blood Count (CBC)

Twenty μl of uncoagulated whole blood were drawn by cardiac puncture in anesthetized mice using needles and syringes coated with 4% sodium citrate (Sigma). CBC analysis was performed by a Hemavet 950 (Drew Scientific, Dallas, TX) or by an IDEXX veterinary reference lab (Markham, ON), using a Sysmex Hematology Analyzer Model XT2000V. We independently verified both methods and similar results were obtained. MULTI-TROL calibration controls (Drew Scientific) were run before each series of Hemavet measurements.

### Bone marrow neutrophil isolation

Bone marrow neutrophils from femurs and tibias were harvested and cleaned as described previously [36]. Briefly, bone marrow was flushed with 10 ml ice-cold minimum essential medium eagle alpha modification (α-MEM) and samples were homogenized gently using a 20-gauge needle and spun down for 10 minutes at 700 xg at 4°C. The pellets were resuspended in 1 ml 1X Dulbecco’s phosphate buffered saline without calcium chloride and magnesium chloride (dPBS-/-) (Sigma), neutrophils were isolated on a discontinuous 82%/65%/55% Percoll gradient (Sigma) prepared in dPBS-/- and centrifuged for 30 min at 1,015xg at 4°C. The neutrophil layer (between 65% and 82%) was washed and counted using a Z1 Coulter Particle Counter (Beckman Coulter, Fullerton, CA). As described previously [37], purity was determined by Romanowsky Diff-Quik staining of methanol-treated air-dried smears according to manufacturer’s instructions (Sigma), and was >90%.

### Isolation of peritoneally-recruited neutrophils

Isolation and enumeration of neutrophils recruited to the peritoneal cavity following intraperitoneal injection of 1 ml 5 nM sodium periodate (Sigma) were performed as previously described [38]. Briefly, 2 to 3 hours after sodium periodate injection, mice were anesthetized using isoflurane and euthanized by cervical dislocation. The abdominal surface was cleaned with 70% ethanol followed by ventral midline incision and skin retraction. Ten ml of sterile ice-cold 1X dPBS-/- were injected into the abdominal cavity without perforating organs, massaged for 5–10 minutes, and fluid was withdrawn. Samples were centrifuged at 700 xg 4°C for 10 min in a fixed angle rotor, washed twice with 10 ml ice-cold 1X dPBS-/-, resuspended in 1 ml dPBS-/- and counted using a Z1 Coulter Particle Counter, as described above. Sample purity measured by Diff-Quik staining was >90%. Mice which were used for isolation of peritoneally-recruited neutrophils were not used for any other experiment (e.g. measurement of bacterial burden, blood glucose, histology or isolation of bone marrow neutrophils) to prevent artefacts associated with acute inflammation.

### *Ex vivo* neutrophil bacterial uptake and killing assays

At the end of the experimental or control treatments, animals were sacrificed and neutrophils were harvested from bone marrow and peritoneum and co-incubated *in vitro* with opsonized *E*. *coli* or *B*. *burgdorferi*. Opsonization was performed with pre-immune serum from congenic normoglycemic mice. After co-incubation, numbers of intact extracellular bacteria (*B*. *burgdorferi*) or colony-forming units (CFUs: *E*. *coli*) relative to input bacterial numbers were measured to assess neutrophil uptake and killing of bacteria. Opsonized bacteria incubated under the same conditions in the absence of neutrophils were used as a control.

*E*. *coli* DH5α was cultured in LB Lennox-broth overnight at 37°C, OD_600_ was measured using an Ultraspec 3000 (Biochrom Ltd., Cambridge, UK), and 3x10^6^ bacteria were opsonized for 30 minutes at 37°C with 5 μl serum from normoglycemic uninfected mice. Opsonized bacteria were incubated with 1x10^6^ neutrophils at a multiplicity of infection (MOI) of 3:1 for 1 hour at 37°C_._ After incubation, bacteria-neutrophil mixtures were diluted 1:10^4^ and 1:10^5^ in dPBS-/- and 100 μl of dilutions were plated in triplicate on LB Lennox-agar plates (Bioshop) and incubated overnight at 37°C. CFUs were enumerated for all replicates and dilutions, and normalized to CFUs for similar dilutions of complement-opsonized input bacteria stored on ice until plating.

*B*. *burgdorferi* (1x10^7^) cultivated to log phase (<7x10^7^/ml) were opsonized at 36°C and 1.5% CO_2_ for 30 minutes in 5 μl blood serum from normoglycemic uninfected C3H/HeN mice. Neutrophils (1x10^6^) were incubated with opsonized bacteria at a MOI of 10:1 and incubated 16 h in Roswell Park Memorial Institute (RPMI) media (Sigma) containing 5% heat-inactivated fetal bovine serum at 36°C and 1.5% CO_2_. Numbers of intact bacteria remaining after overnight incubation were counted with a Petroff-Hausser counting chamber (Hausser Scientific, Horsham, PA) and normalized to counts for complement-opsonized input bacteria stored at 4°C until uptake counting was performed. *B*. *burgdorferi* viability was determined by LIVE-DEAD staining (ThermoFisher Scientific), performed according to manufacturers’ instructions.

### Statistical analyses

Statistical analyses of all measured parameters in experimental groups were performed using GraphPad Prism v6.0 graphing and statistical analysis software (GraphPad Software, La Jolla, CA). Normally distributed data were analyzed using two-way ANOVA with Holm-Sidak post-tests or paired t-tests. Non-normally distributed data were log-transformed and analyzed by one-way Kruskal-Wallis ANOVA with Dunn’s post-tests, or by two-tailed Mann-Whitney t-tests. Normality testing was performed using the D'Agostino & Pearson omnibus normality test. *P* values of <0.05 were considered significant.

## Results

### Hyperglycemic mouse models of *B*. *burgdorferi* infection

Initial studies to determine if hyperglycemia affected *B*. *burgdorferi* dissemination and clearance were performed in the most widely reported mouse model of insulin-insufficient diabetes, low dose streptozotocin (STZ)-treated C57BL/6 mice [39]. STZ is an antibiotic which induces irreversible insulin insufficiency by killing pancreatic β-cells [26]. C57BL/6 mice are readily infected by *B*. *burgdorferi* but are resistant to infection-induced inflammatory pathologies in joints (arthritis) and heart (carditis) [2]. In subsequent experiments, hyperglycemia was induced by STZ treatment in the arthritis- and carditis-susceptible C3H/HeN mouse model of Lyme disease, to measure effects of hyperglycemia on Lyme disease pathology. Induction of hyperglycemia in C3H/HeN mice requires more extended treatment with STZ [26], and the effects of hyperglycemia on immune responses to bacterial infection in this background have not been characterized. To exclude potential hyperglycemia-independent effects of STZ treatment, infections were also conducted in C57BL/6-derived insulin-insufficient heterozygote Akita mice, which carry a dominant-negative mutation in the insulin 2 gene resulting in spontaneous emergence of type 1 diabetes in young adult animals [31,40]. Infections were also conducted in age-matched littermates which did not carry the Akita mutation. Due to variable time to onset of hyperglycemia in Akita animals, mice used for Akita experiments were from a broader age range than those used in STZ experiments. Mouse strains used in this study and their characteristics are summarized in Table 1.

**Fig 1:**
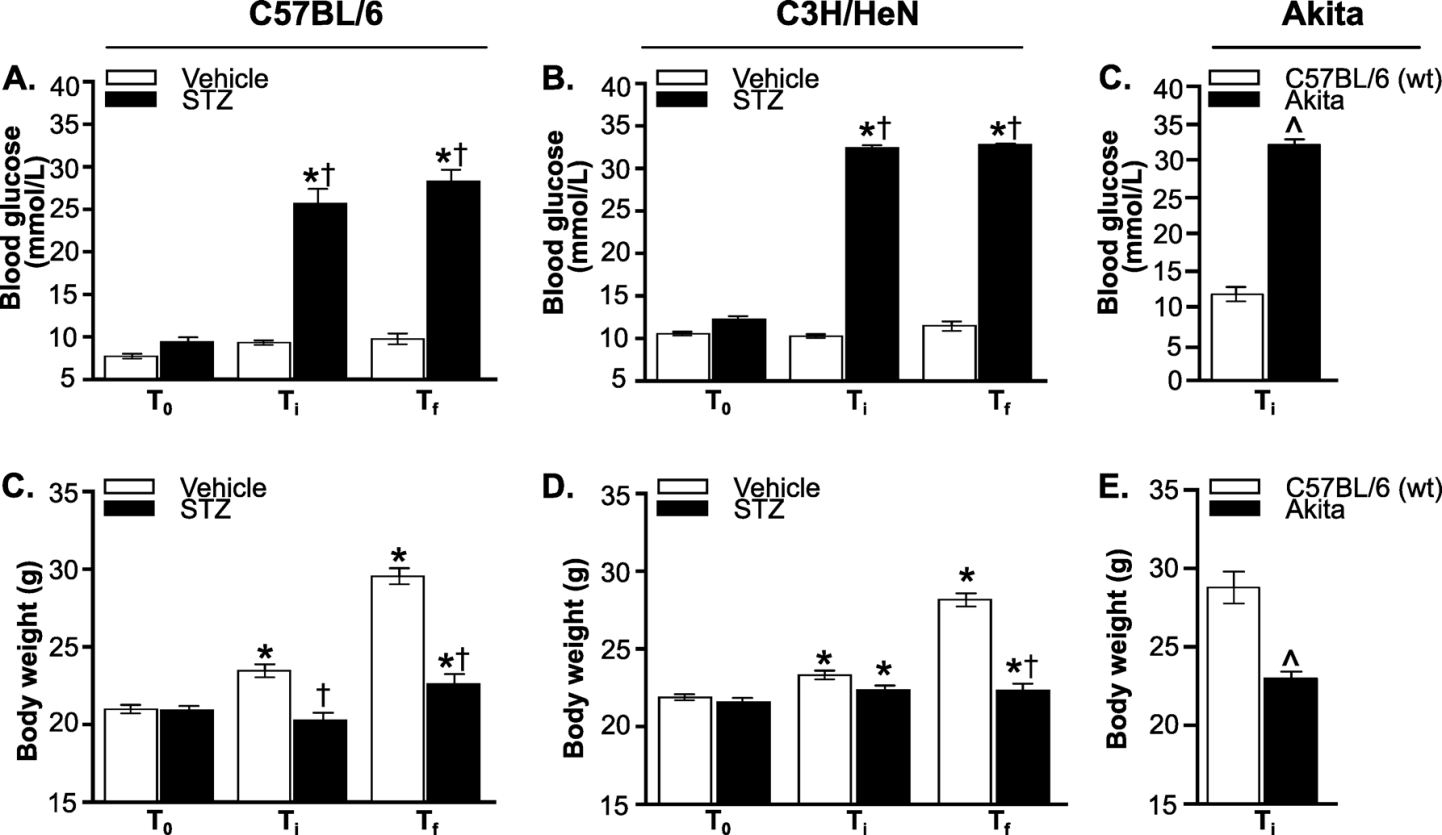
Hyperglycemic mouse models of *B*. *burgdorferi* infection. (A-C) Non-fasting blood glucose levels in STZ- and vehicle-treated C57BL/6 (A) and C3H/HeN (B) mice, and in Akita mice and age-matched wild type C57BL/6 mice (C). (D-F) Body weight of STZ- and vehicle-treated C57BL/6 (D) and C3H/HeN (E) mice, and of Akita mice and age-matched wild type C57BL/6 mice (F). T_0_ stands for baseline values (before STZ treatment), T_i_ corresponds to time of infection, and T_f_ stands for time of sacrifice. N = 17–21 mice per group. Statistical analysis: (A-B, D-E) Two-way ANOVA with Holm-Sidak post-tests. (C, F) Two-tailed t-test. * indicates p<0.05 vs. T_0_ within group; † indicates p<0.05 vs. Vehicle within time point. ^ indicates p<0.05 vs wild-type.

To induce hyperglycemia, we treated 5-week old mice with 40 μg of STZ for 5 (C57BL/6) or 7 (C3H/HeN) consecutive days, followed by a 5 day “washout” period to ensure that STZ was no longer present in tissues at the time of infection [26]. Age- and strain-matched control mice were treated with vehicle only (buffer without STZ). Mice were infected with a B31 5A4-derived *B*. *burgdorferi* strain [33] or mock-infected with cultivation medium alone, and sacrificed four weeks after infection. STZ treatment increased non-fasting blood glucose levels to >25 mmol/L in both C57BL/6 and C3H/HeN mice, similarly to previously reported values [41]. Thus, STZ-treated mice were hyperglycemic. Blood glucose levels were comparable at time of infection (T_i_) and sacrifice (T_f_) (Fig 1A and 1B), indicating that a stable state of hyperglycemia was maintained over the course of infection and that regeneration of β-cells likely did not occur [42]. Blood glucose levels in all normoglycemic infected mice also remained stable throughout the experiment, indicating that *B*. *burgdorferi* infection did not affect blood glucose (Fig 1A and 1B**)**. Heterozygous Akita mice were significantly more hyperglycemic than STZ-treated C57BL/6 counterparts, but blood glucose levels in normoglycemic control animals in STZ and Akita experiments also differed significantly (p<0.05, compare Ti values in Fig 1A and 1C). This was possibly because the age ranges of mice used in these experiments differed, or because the C57BL/6 strains used in STZ and Akita experiments originated with different suppliers (Table 1: Charles River BL/6 NCrl for STZ experiments, BL/6J Jackson Labs for Akita experiments). Body weight of STZ-treated and Akita heterozygotes, but not vehicle-treated mice or age-matched wild-type Akita littermates, was significantly reduced at the time of sacrifice compared to baseline (Fig 1D–1F), consistent with previous reports of reduced weight gain during maturation to adulthood in hyperglycemic mice [30]. As for blood glucose, body weights differed significantly in hyperglycemic Akita and STZ-treated C57 mice and in normoglycemic controls (p<0.05, compare Ti values in Fig 1D and 1F). Together, these data indicated that STZ treatment and Akita heterozygosity induced hyperglycemia.

### More widespread *B*. *burgdorferi* colonization and reduced bacterial clearance in tissues of hyperglycemic mice

**Fig 2:**
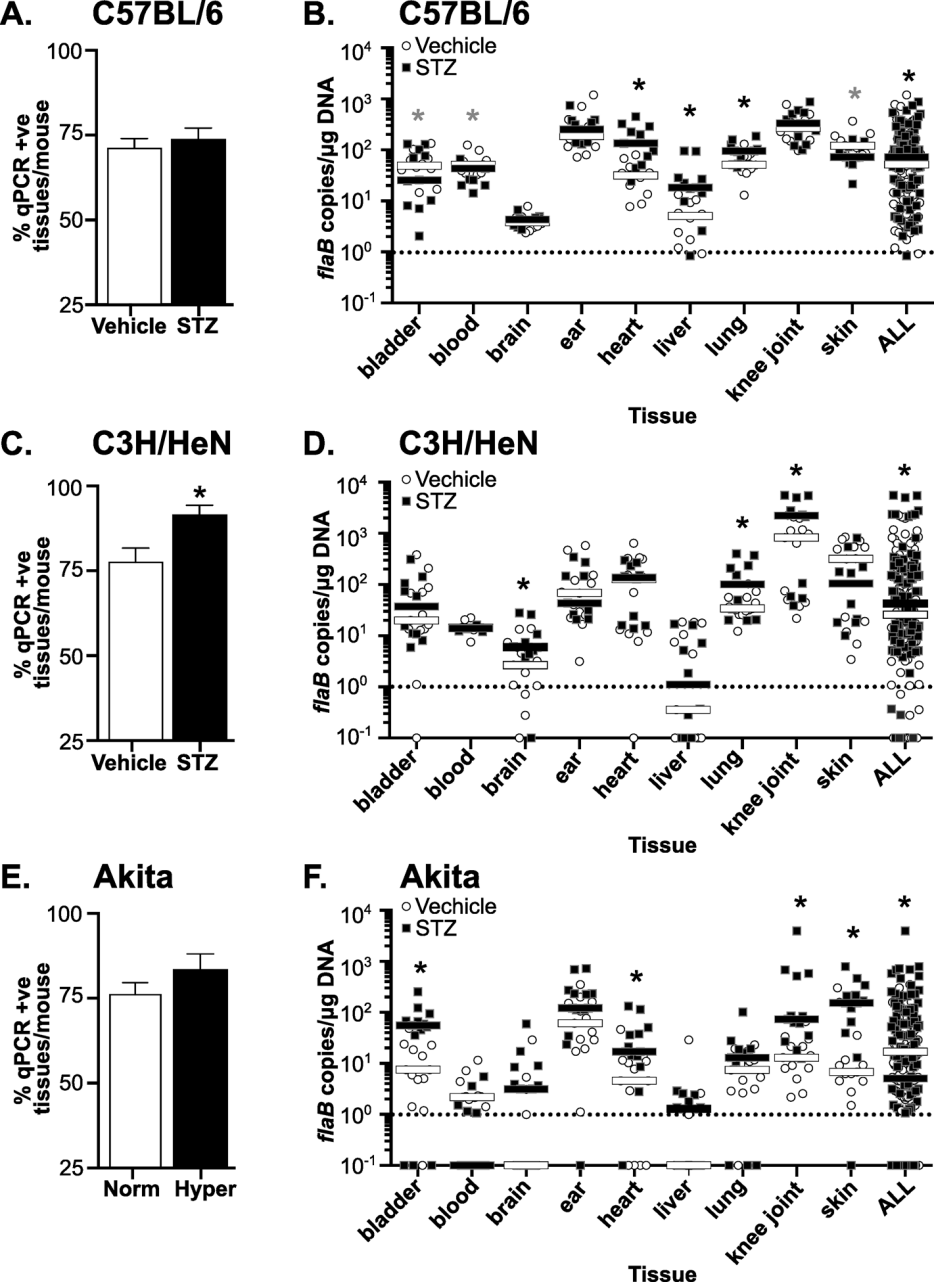
*B*. *burgdorferi* tissue colonization and bacterial DNA copy number in hyperglycemic and normoglycemic mice. (A, C and E) Percentage of tissues/mouse positive for *B*. *burgdorferi flaB* DNA in infected normoglycemic and hyperglycemic mice at 4 weeks post-infection. Percentage of qPCR-positive tissues/mouse in C57BL/6 (A), C3H/HeN (C), and Akita (E) mice are shown. (B, D and F) Median *B*. *burgdorferi flaB* copy number/μg total DNA in indicated tissues and in all tissues combined (ALL) in infected normoglycemic and hyperglycemic mice at 4 weeks post-infection. Shown are individual values and medians (bars) in tissues of C57BL/6 (B), C3H/HeN (D) and Akita (F) mice. Values are plotted on a log scale to facilitate visualization of a large range of values. Dotted lines in each graph indicate the cutoff point (1 *flaB* copy/μg DNA) below which tissues were considered negative. Statistical analysis: Kruskal-Wallis ANOVA with Dunn’s post-test. For all panels, N = 10–13 mice per experimental group and strain. Fold differences in medians are summarized in Table 2. * indicates p<0.05 vs. normoglycemic controls.

To detect *B*. *burgdorferi* DNA in tissues of infected mice and to determine if hyperglycemia altered tissue bacterial burden, samples were harvested from mice at 4 weeks post-infection (after 5–6 weeks of sustained hyperglycemia). The examined tissues were bladder, blood, brain, ear, heart, liver, lung, knee joint (patella) and ventral thoracic skin. Previous studies have reported that *B*. *burgdorferi* can be cultivated and detected by quantitative PCR (qPCR) in all of these tissues [2–4,43–46]. Heart, skin, joint, bladder and ear are among the most common targets examined in murine pathogenesis studies, because these tissues often display higher *B*. *burgdorferi* burden and are the sites of disease pathology in mice (heart, joint). Tissues such as brain and liver are much less commonly positive for these bacteria, but can still be infrequently colonized. We collected samples from tissues typically targeted by *B*. *burgdorferi* (bladder, blood, ear, heart, knee joint and ventral thoracic skin), tissues that exhibit physiological dysfunction in hyperglycemia (liver, brain) [47,48], and tissues where hyperglycemia is associated with impaired immune responses to bacterial infection (lung) [49]. *B*. *burgdorferi* DNA copy number was quantified by measuring absolute copy number of bacterial *flaB* DNA sequence per microgram of extracted DNA by qPCR, as previously described [32,33] (Fig 2). We did not determine viability of *B*. *burgdorferi* isolated from individual organs.

We first assessed whether hyperglycemia affected the extent of *B*. *burgdorferi* dissemination to and colonization of tissues by calculating the average percentage of tissues per mouse in which at least one *flaB* copy/μg total extracted DNA was detected (Fig 2A, 2C and 2E). The percentage of tissues/mouse which were positive for *flaB* DNA was calculated by assigning a score of 1 to each tissue with at least 1 *flaB* copy/μg DNA and a score of 0 to tissues where <1 *flaB* copy was measured. The sum of these scores for all tissues in each mouse (bladder, blood, brain, ear, heart, liver, lung, knee joint and skin) was divided by the total number of tissues tested in each mouse to determine the percentage of tissues/mouse which were *flaB*-positive. The mean percentage of *flaB*-positive tissues per mouse was somewhat elevated in STZ-treated C57BL/6 mice (Fig 2A) and hyperglycemic Akita heterozygotes (Fig 2C), but not significantly. However, significantly more tissues per mouse were *flaB*-positive in STZ-treated C3H/HeN mice (Fig 2B), suggesting a more widespread colonization in this mouse strain in the context of hyperglycemia. Overall, hyperglycemia was associated with a significant 11% average increase in the number of qPCR-positive tissues per mouse across all mouse strains (p<0.05).

We next compared median *B*. *burgdorferi flaB* DNA copy numbers in tissues of hyperglycemic and normoglycemic mice (Fig 2B, 2D and 2F). Median values were compared because copy number values in most tissues and experimental groups were not normally distributed. Values shown in Fig 2B, 2D and 2F were plotted on log scales to facilitate graphing of a wide range of copy number values on the same plots. As expected, *flaB* copy number in liver and brain were lower than in other tissues for normoglycemic mice (Fig 2B, 2D and 2F). Unexpectedly, *flaB* copy numbers in skin and knee joints, and in all tissues combined (ALL) differed significantly in vehicle-treated C57BL/6 mice compared to wild-type Akita C57BL/6 littermates (p<0.05, compare Fig 2B and 2F). This indicated that in addition to strain/supplier-origin-specific differences in blood glucose levels and body weight in these animals, bacterial burden was also affected.

In hyperglycemic STZ-treated C57BL/6 mice, median copy number for all tissues combined was significantly increased by 1.4-fold compared to normoglycemic controls (Fig 2B, Table 2: ALL). As observed in hyperglycemic C57BL/6 mice, overall tissue copy number was significantly increased by 1.7-fold in hyperglycemic STZ-treated C3H/HeN mice compared to normoglycemic controls (Fig 2D, Table 2: ALL). Copy number across all tissues was also significantly elevated by 3.4-fold in hyperglycemic Akita mice (Fig 2F, Table 2: ALL). Across all mouse strains combined, copy number for all tissues was 1.6-fold greater in hyperglycemic than normoglycemic mice. Tissues where copy number was consistently and significantly elevated in hyperglycemia were brain, heart, liver, lung and knee joint, where median copy numbers were 1.9 to 2.8-fold greater across all mouse strains (Table 2). Together, these results indicated that hyperglycemia was associated with more widespread *B*. *burgdorferi* colonization and reduced clearance of bacterial DNA debris.

**Table 2:** Median fold-differences in *B*. *burgdorferi* DNA copy number in hyperglycemic vs normoglycemic mice.

Tissue	Mouse strain			
	C57BL/6	C3H/HeN	Akita	All mouse strains
Bladder	**-5.0**^a^	+3.7	**+7.4**^*a*^	+2.5
Blood	**-2.4**^*a*^	-1.1	-1.5	**-1.2**^*a*^
Brain	+1.2	**+2.8**^*a*^	+3.2	**+1.9**^*a*^
Ear	+1.3	+1.6	+2.0	+1.7
Heart	**+4.1**^*a*^	1.0	**+3.7**^*a*^	**+2.3**^*a*^
Liver	**+3.9**^*a*^	+4.8	+1.3	**+2.4**^*a*^
Lung	**+1.9**^*a*^	**+4.5**^*a*^	+1.8	**+1.9**^*a*^
Knee joint (patella)	+1.2	**+2.8**^*a*^	**+5.7**^*a*^	**+2.8**^*a*^
Skin	**-1.7**^*a*^	-1.4	**+22.7**^*a*^	-1.3
**ALL tissues**	**+1.4**^*a*^	**+1.7**^*a*^	**+3.4**^*a*^	**+1.6**^*a*^

^*a*^ indicates p<0.05 hyperglycemic vs normoglycemic, Kruskal-Wallis ANOVA with Dunn’s post-test

### Reduced incidence of arthritis but not carditis in *B*. *burgdorferi*-infected hyperglycemic mice

Lyme carditis is an inflammatory condition, which is characterized in susceptible mouse strains (C3H, but not C57BL/6 mice) by increased leukocyte infiltration and fibroblast proliferation [25,50]. Prolonged hyperglycemia can cause reduced cellular density in the heart resulting from accumulation of excess extracellular matrix [51]. To distinguish between possible effects of hyperglycemia (hypocellularity) and *B*. *burgdorferi*-induced carditis (hypercellularity), we adapted a nuclei-counting method for measuring infiltration of inflammatory cells in multifocal cardiac inflammation that enabled us to quantitatively distinguish these conditions [35]. Numbers of nuclei in five 100 mm^2^ regions of interest in two-three matched hematoxylin- and eosin-stained sagittal sections per heart were enumerated using a counting grid. Nuclei were counted in each atrium and ventricle and the heart apex, and the majority of tissue included in each region of interest was derived from the myocardium.

**Fig 3:**
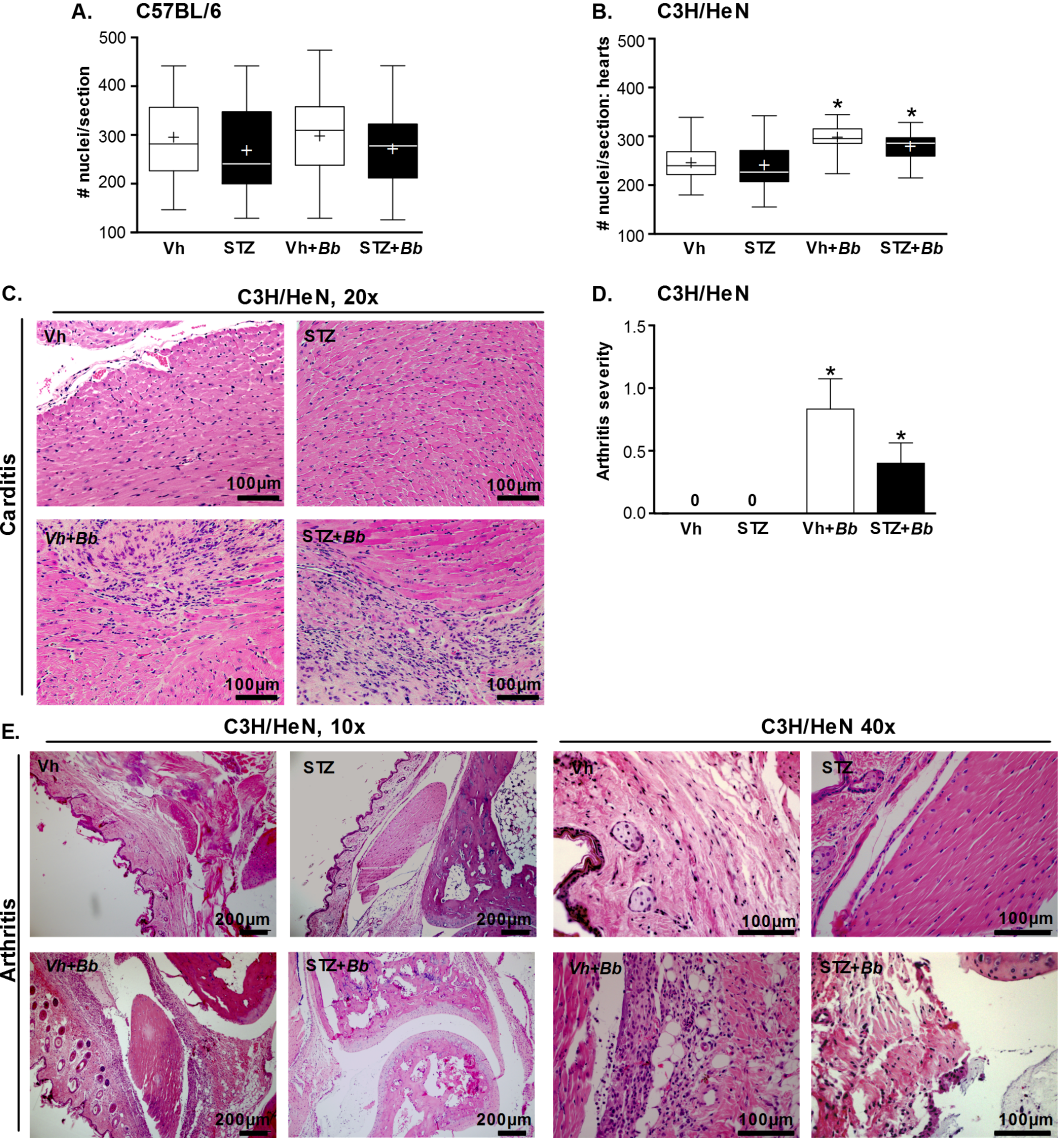
*B*. *burgdorferi*-induced carditis and arthritis in hyperglycemic and normoglycemic mice. (A-B) Cardiac cellularity in C57BL/6 (A) and C3H/HeN (B) mice. Experimental groups: normoglycemic mock-infected (Vh), hyperglycemic mock-infected (STZ), normoglycemic infected (Vh+*Bb*), hyperglycemic infected (STZ+*Bb*). The numbers of nuclei in five 100 mm^2^ regions of interest in 2–3 matched H&E-stained sagittal sections per heart were enumerated using a counting grid. Nuclei were counted in each atrium and ventricle and the heart apex, and the majority of tissue included in each region of interest was derived from the myocardium. Summary values are shown for the average numbers of nuclei/section/mouse. Tukey box plots represent the 25–75% range, line and plus symbols (+) correspond to medians and means, respectively, and error bars span minimum to maximum values. N = 11–15 mice per group. Statistics: Two-way ANOVA with Holm-Sidak post-test. * indicates p<0.05 vs mock-infected within vehicle- or STZ-treated groups. (C) Representative H&E-stained sagittal heart sections of C3H/HeN mice. Scale bar: 100 μm. (D) Arthritis scoring in C3H/HeN mice. Scoring of arthritis severity was performed in a blinded fashion by a pathologist using the following scoring system: 0: no pathology, 1: mild pathology, 2: severe pathology. N = 10–11 mice/group. Shown are mean ±SEM severity scores. Statistics: Kruskal-Wallis ANOVA with Dunn’s post-test. * indicates p<0.05 vs. mock-infected within vehicle- or STZ-treated groups. (E) Representative H&E-stained tibiotarsal joint sections of C3H/HeN mice. Scale bars: 100–200 μm.

No significant changes in cellularity measured by this method were detected in hyperglycemic and normoglycemic carditis-resistant C57BL/6 (Fig 3A). In carditis-sensitive C3H/HeN mice, hypercellularity indicative of carditis was significantly elevated in both hyperglycemic and normoglycemic infected animals. However, STZ treatment, which did not alter bacterial DNA copy number in the hearts of C3H/HeN mice, had no additional effect on carditis severity (Fig 3B). Representative histology sections of C3H/HeN mice are shown in Fig 3C. Since Akita mice are C57BL/6-derived, and therefore likely resistant to both carditis and arthritis, we did not perform histological analysis on samples from these mice. Collectively, these results indicated that *B*. *burgdorferi* infection resulted in inflammation in the heart of C3H/HeN, but not C57BL/6 mice, and that hyperglycemia did not alter the levels of cardiac inflammation in either mouse strain. Since bacterial DNA burden was not elevated in hearts of hyperglycemic C3H/HeN mice, we concluded that hyperglycemia did not affect carditis severity in a fashion that was independent of bacterial clearance.

In contrast to heart, bacterial copy number was significantly greater in knee joints of hyperglycemic C3H/HeN animals (Fig 2D). Arthritis is a prominent pathology observed following *B*. *burgdorferi* infection in juvenile (3 week-old) C3H mice, and is secondary to infiltration of neutrophils and other leukocytes into joints [2]. To investigate the effect of hyperglycemia on arthritis, we induced hyperglycemia by STZ treatment in 3-week old C3H/HeN mice, followed by *B*. *burgdorferi* infection at just under 5 weeks of age. As for the older mice used in all other experiments in our study, STZ treatment induced significant hyperglycemia in 3-week old mice (>30 mmol/L; p<0.05 compared to vehicle-treated controls; data not shown). Four weeks after infection (5–6 weeks of sustained hyperglycemia), arthritis in tibiotarsal joints was scored by a murine veterinary pathologist blinded to the identity of experimental samples (Fig 3D). *B*. *burgdorferi* infection caused arthritis in both normoglycemic and hyperglycemic mice, and arthritis was absent in mock-infected controls (Fig 3D). Arthritis incidence was significantly decreased in hyperglycemic infected animals compared to normoglycemic counterparts (40.0 vs 58.3% incidence; p<0.05), but reduction in arthritis severity in hyperglycemic mice was not significant (Fig 3D). Representative joint histology images are displayed in Fig 3E. Thus, although bacterial burden was significantly elevated in the joints of hyperglycemic C3H/HeN mice, inflammatory pathology was less frequently observed.

### Impaired *B*. *burgdorferi* killing by activated neutrophils isolated from hyperglycemic mice

Neutrophils contribute to control of *B*. *burgdorferi* burden and inflammatory pathology in joints [23], and their function is often impaired in the context of hyperglycemia [13–18]. This prompted us to determine if the ability of neutrophils from hyperglycemic mice to control bacterial survival *ex vivo* was altered. To determine whether the bactericidal function of neutrophils toward *B*. *burgdorferi* was disrupted in hyperglycemia, we measured uptake and killing of *B*. *burgdorferi* and a control bacterium (*E*. *coli*) [10] by non-activated neutrophils isolated from bone marrow, as well as activated neutrophils recruited to the peritoneum in response to acute inflammatory stimulus (sodium periodate). Since variable age of hyperglycemia onset in C57BL/6-derived Akita heterozygotes made age-matching challenging, we performed these experiments only in STZ-treated C57BL/6 and C3H/HeN mice, where age of animals was identical and inter-subject variation was minimized.

**Fig 4:**
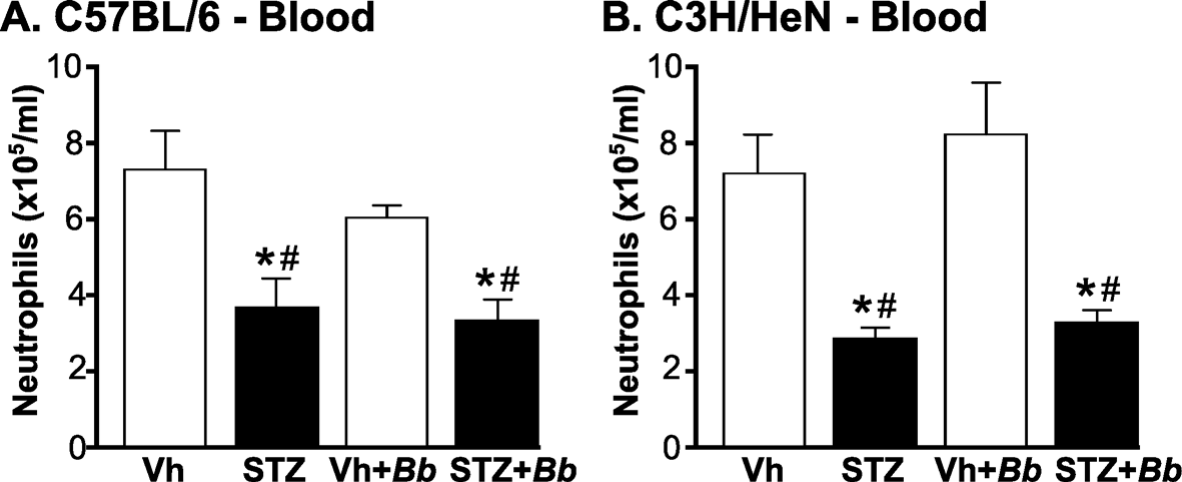
Neutropenia in *B*. *burgdorferi*-infected and mock-infected hyperglycemic mice. (A, B) Mean ±SEM numbers of circulating neutrophils in blood of C57BL/6 (A) and C3H/HeN mice (B). Neutrophils were enumerated by particle counting with a Coulter counter in blood drawn by cardiac puncture from *B*. *burgdorferi*-infected and mock-infected mice at 4 weeks post-infection (5–6 weeks of sustained hyperglycemia). N = 6–9 mice/group. Experimental groups: Normoglycemic mock-infected (Vh), hyperglycemic mock-infected (STZ), normoglycemic *B*. *burgdorferi*-infected (Vh+*Bb*), hyperglycemic *B*. *burgdorferi*-infected (STZ+*Bb*). Statistical analysis: Two-way ANOVA with Holm-Sidak post-tests. * indicates p<0.05 vs. Vehicle; # indicates p<0.05 vs. normoglycemic *B*. *burgdorferi*-infected (Vh+*Bb*) mice.

We first determined numbers of peripheral neutrophils in blood of C3H/HeN and C57BL/6 mice at 4 weeks post-infection after 5–6 weeks of sustained hyperglycemia (Fig 4). Neutrophil counts in mice were significantly reduced in both hyperglycemic C57BL/6 and C3H/HeN mice independent of infection status (Fig 4A and 4B). Importantly, significant neutropenia was present in hyperglycemic mice infected with *B*. *burgdorferi* (Fig 4A and 4B), indicating that *B*. *burgdorferi* infection was not sufficient to overcome deficits in neutrophil production and/or mobilization in bone marrow of diabetic animals.

**Fig 5:**
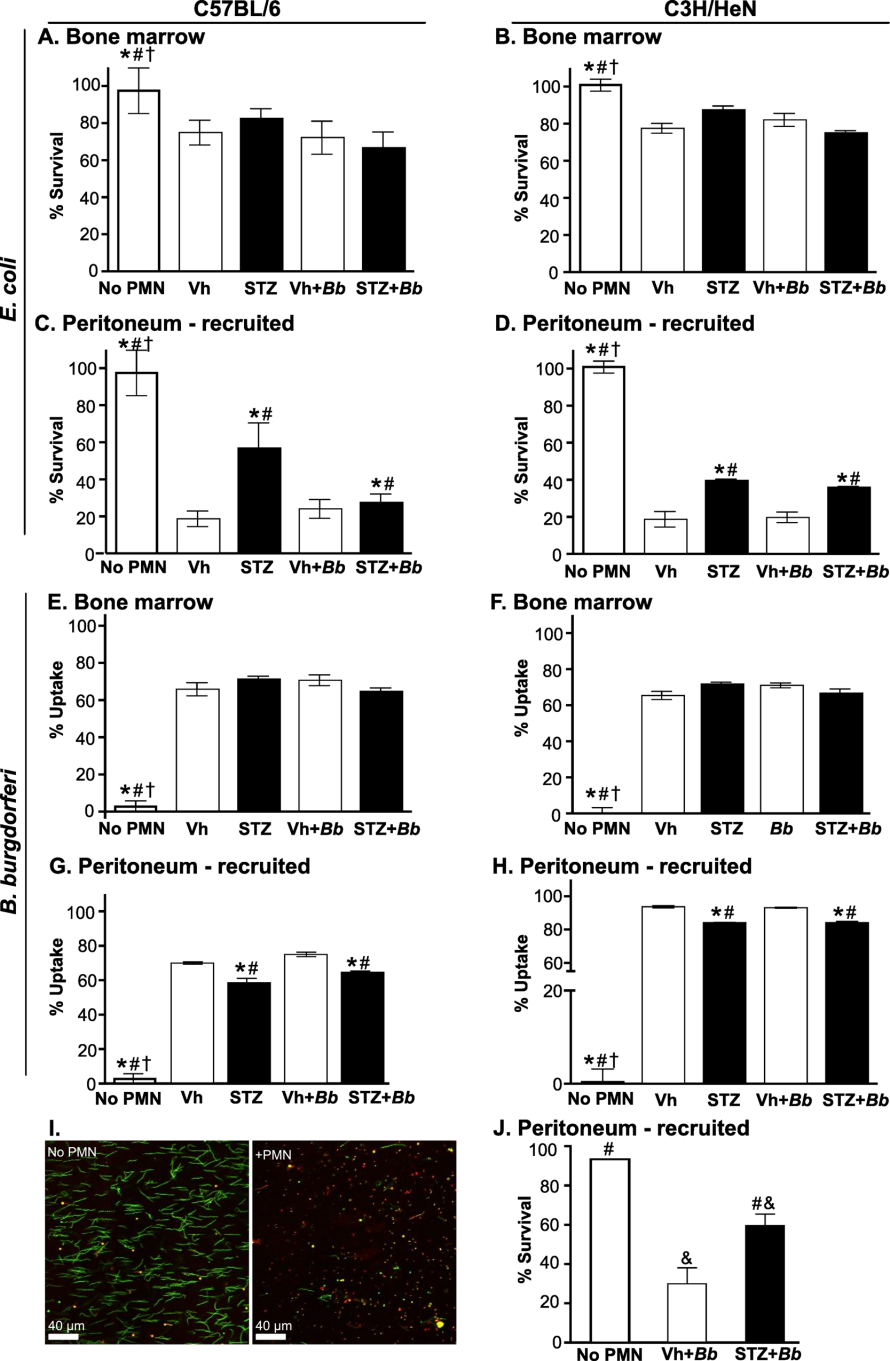
Bacterial uptake and survival following co-incubation with neutrophils isolated from hyperglycemic and normoglycemic mice. Bone marrow (A-B and E-F) or peritoneally-recruited (C-D and G-J) neutrophils were isolated from *B*. *burgdorferi*-infected and mock-infected C57BL/6 (A, C, E, G) and C3H/HeN (B, D, F, H-J) mice at 4 weeks post-infection (5–6 weeks of sustained hyperglycemia), and coincubated with complement-opsonized *E*. *coli* (A-D) or *B*. *burgdorferi* (E-J). *E*. *coli* survival (A-D) and *B*. *burgdorferi* uptake by neutrophils (E-H) were measured by comparing numbers of *E*. *coli* CFUs and intact *B*. *burgdorferi* following neutrophil co-incubation with values for complement-opsonized input bacteria. (I-J) *B*. *burgdorferi* killing was measured by LIVE-DEAD staining. Panel I shows sample images for bacteria incubated in the absence (no PMN) and presence (+PMN) of neutrophils. Panel J shows quantification of *B*. *burgdorferi* killing. Summary values: mean ±SEM. N = 4–6 mice/group. Experimental groups: No neutrophil (PMN) control: opsonized bacteria incubated in absence of neutrophils, normoglycemic mock-infected (Vh), hyperglycemic mock-infected (STZ), normoglycemic *B*. *burgdorferi*-infected (Vh+*Bb*), hyperglycemic *B*. *burgdorferi*-infected (STZ+*Bb*). Statistical analysis: Two-way ANOVA with Holm-Sidak post-tests. * indicates p<0.05 vs. Vehicle; # indicates p<0.05 vs. normoglycemic *B*. *burgdorferi*-infected (Vh+*Bb*) mice; † indicates p<0.05 vs. hyperglycemic mock-infected mice (STZ); & indicates p<0.05 vs no PMN control (panel J only).

We next examined the ability of neutrophils harvested from C57BL/6 (Fig 5A, 5C, 5E and 5G) and C3H/HeN (Fig 5B, 5D, 5F and 5H–5J) mice after 4 weeks of *B*. *burgdorferi* infection (5–6 weeks of sustained hyperglycemia) to control uptake and survival of *B*. *burgdorferi* opsonized with pre-immune serum (Fig 5E–5J). Pre-immune serum was used to investigate the effects of hyperglycemia on neutrophil function independent of its effects on adaptive immune responses, and to maintain consistency with the methods of a previous study measuring effects of hyperglycemia on neutrophil killing of *E*. *coli* [18]. Opsonization in this context therefore refers to exposure of bacteria to serum complement proteins [52].

Uptake was measured by determining the percentage of *B*. *burgdorferi* which remained following co-incubation with neutrophils, compared to mock-treated bacteria incubated without neutrophils. *B*. *burgdorferi* survival was determined by LIVE-DEAD staining, which identifies cells with permeable membranes. Killing of pre-immune serum-opsonized *E*. *coli*, which is impaired in neutrophils from hyperglycemic mice [18] was also measured (Fig 5A–5D), both as a control, and to determine if ongoing *B*. *burgdorferi* infection altered the ability of neutrophils to kill other bacteria *ex vivo*. Hyperglycemia primarily affects the bactericidal function of activated neutrophils isolated from the circulation and tissues, possibly because hyperglycemia dysregulates neutrophil transition to the fully activated state required for bactericidal activities [7–10]. To determine whether control of bacterial viability *ex vivo* depended on tissue-specific activation factors encountered during recruitment (peritoneal exudate) or systemic factors promoting neutrophil activation in bone marrow, we performed assays with neutrophils harvested from bone marrow (Fig 5A, 5B, 5E and 5F) as well as neutrophils obtained from peritoneal exudates following acute sodium periodate treatment (Fig 5C and 5D and 5G–5J).

Neither hyperglycemia nor *B*. *burgdorferi* infection affected *B*. *burgdorferi* uptake (Fig 5E and 5F) or *E*. *coli* survival (Fig 5A and 5B) following co-incubation with bone marrow neutrophils. By contrast, significantly more *E*. *coli* survived following incubation with peritoneal neutrophils from hyperglycemic C57BL/6 and C3H/HeN mice than normoglycemic controls (Fig 5C and 5D). Interestingly, *E*. *coli* survival following incubation with peritoneal neutrophils isolated from both normoglycemic and hyperglycemic infected C57BL/6 mice was similar to mock-infected normoglycemic animals, but was significantly lower than in mock-infected STZ-treated animals (Fig 5C). *B*. *burgdorferi* uptake was also impaired in hyperglycemic animals (Fig 5G and 5H). Approximately 2-fold more *B*. *burgdorferi* survived following co-incubation with peritoneal neutrophils from hyperglycemic C3H mice, as determined by LIVE-DEAD staining (Fig 5I and 5J). These data confirmed previous reports that hyperglycemia impairs the ability of activated neutrophils to kill *E*. *coli* [18], and indicated that hyperglycemia also inhibits the ability of activated neutrophils to phagocytose and kill *B*. *burgdorferi*.

## Discussion

These studies demonstrated that hyperglycemia induces neutropenia and inhibits neutrophil *B*. *burgdorferi* killing in mice, which are associated with impaired bacterial clearance in multiple tissues and protection against Lyme arthritis. Type I and Type II diabetes affect susceptibility to and outcomes of infection with diverse pathogens in both humans and animal models [9,11–18,53–55]. Type I and II diabetes have different effects on immune function, due to the presence of comorbid obesity and insulin resistance in Type II diabetes [56]. However, one of the most prominent immune deficiencies arising from hyperglycemia itself is impaired activation of neutrophils, which is associated with reduced phagocytosis, bacterial killing and bacterial clearance from tissues [13,15–18,57–61]. Our data indicate that *B*. *burgdorferi* is another member of the growing list of bacteria for which hyperglycemia-dependent neutrophil dysfunction has been associated with reduced bacterial killing and clearance in infection.

We did not investigate numbers of cultivatable *B*. *burgdorferi* in tissues, and therefore do not know if clearance inhibition in mice reflected deficits in killing of bacteria or in removal of their debris. Furthermore, we estimated neutrophil *B*. *burgdorferi* killing *ex vivo* using LIVE-DEAD staining, which identifies bacteria with membranes that are permeable to propidium iodide, and not viability per se. Nevertheless, the hyperglycemia-associated ~2-fold reduction in neutrophil *B*. *burgdorferi* killing measured by LIVE-DEAD staining in *ex vivo* experiments was very similar to the ~1.7-fold reduction in clearance of *B*. *burgdorferi* DNA from tissues of hyperglycemic mice. Together, these observations suggest that hyperglycemia-dependent deficits in control of bacterial burden *in vivo* were at least partly due to inhibition of neutrophil *B*. *burgdorferi* killing.

Our finding that hyperglycemia affected the bacterial uptake function of neutrophils recruited to tissues but not of bone marrow neutrophils also agrees with previous reports that hyperglycemia primarily affects the function of circulation and tissue neutrophils, likely due to dysregulation of neutrophil transition to the fully activated state required for bactericidal activities [15–18,57]. In addition, across all mouse strains and conditions used in our studies, hyperglycemia resulted in more widespread *B*. *burgdorferi* colonization and reduced clearance of *B*. *burgdorferi* debris both in typical targets of this bacterium (heart and joint), but also in organs frequently affected by hyperglycemia (brain, lung and liver) [3,47,49]. Finally, we found that chemically-induced hyperglycemia in two different mouse strains and genetically-induced hyperglycemia were all associated with reduced *B*. *burgdorferi* clearance. Collectively, these data suggest that impaired *B*. *burgdorferi* clearance is associated with hyperglycemia itself, and not with hyperglycemia-independent effects of streptozotocin, the Akita mutation, or mouse strain-specific differences in immune function.

Impaired bacterial phagocytosis and killing in hyperglycemia have been associated with reduced production of lysosomal enzymes, bactericidal/permeability-increasing protein (BPI), and reactive oxygen species, reduced production and activation of elastase, reduced neutrophil extracellular trap (NET) formation and degranulation, as well as glycation-dependent hindrance of C3 binding to bacteria during opsonophagocytosis [18,62–65]. Our studies did not investigate the specific *B*. *burgdorferi* phagocytic and killing activities which were disrupted in neutrophils from hyperglycemic mice. Since *B*. *burgdorferi* is protected from the cytotoxic effects of the neutrophil respiratory burst *in vivo* and *in vitro* [66–71], it is most likely that inhibition of other neutrophil activities is responsible for reduced *B*. *burgdorferi* killing in hyperglycemia, such as reductions in NET formation, C3-dependent phagocytosis and production and activation of BPI and elastase [66,72–76].

Despite reduced clearance of *B*. *burgdorferi* from joints, the incidence of the most prominent neutrophil-based response to infection with this pathogen, Lyme arthritis, was unexpectedly reduced. Neutrophils play an important role in the immunopathology of Lyme arthritis and contribute to, but are not essential for, control of bacterial burden in joints [19,20,22–24]. Differences in Type I interferon (IFN)-dependent neutrophil recruitment to joints are associated with differences in arthritis severity in C3H and C57 mouse strains, which respectively develop severe and mild arthritis in response to *B*. *burgdorferi* infection [2,23,77]. Therefore, it is possible that in our studies neutropenia and impaired neutrophil activation in hyperglycemia protected joints from neutrophil-dependent tissue damage in response to *B*. *burgdorferi*.

The studies reported here demonstrated that hyperglycemia impairs neutrophil responses to *B*. *burgdorferi* and clearance of these bacteria from multiple tissues, and affects the pathological outcomes of *B*. *burgdorferi* infection. While much work remains to determine the mechanisms by which hyperglycemia disrupts immune responses to *B*. *burgdorferi*, these findings suggest that investigating the potential effects of comorbid diabetes on susceptibility to and outcomes of *B*. *burgdorferi* infection in humans may be warranted.
